# Rickettsiaceae and Anaplasmataceae infections in *Ixodes ricinus* ticks from urban and natural forested areas of Poland

**DOI:** 10.1186/1756-3305-7-121

**Published:** 2014-03-24

**Authors:** Renata Welc-Falęciak, Maciej Kowalec, Grzegorz Karbowiak, Anna Bajer, Jerzy M Behnke, Edward Siński

**Affiliations:** 1 Department of Parasitology, Faculty of Biology, University of Warsaw, Miecznikowa 1, 02-096 Warsaw, Poland; 2 W. Stefański Institute of Parasitology of the Polish Academy of Sciences, Twarda 51/55, Warsaw, Poland; 3 School of Life Sciences, University Park, University of Nottingham, Nottingham NG7 2RD, UK

**Keywords:** *Ixodes ricinus*, Tick, *Anaplasma*, *Rickettsia*, *Ehrlichia*, Neoehrlichia, Natural habitats, Urban habitats, Tick density, Prevalence

## Abstract

**Background:**

*Ixodes ricinus* is a major vector for a range of microbial pathogens and the most prevalent and widely distributed tick species on the European continent, occurring in both natural and urban habitats. Nevertheless, little is known about the relative density of ticks in these two ecologically distinct habitats and the diversity of tick-borne pathogens that they carry.

**Methods:**

We compared densities of questing *I. ricinus* nymphs and adults in urban and natural habitats in Central and Northeastern Poland, assessed the prevalence and rate of co-infection with *A. phagocytophilum, Rickettsia, Ehrlichia* and ‘*Ca.* Neoehrlichia spp.’ in ticks, and compared the diversity of tick-borne pathogens using molecular assays (PCR).

**Results:**

Of the 1325 adults and nymphs, 6.2% were infected with at least one pathogen, with 4.4%, 1.7% and less than 0.5% being positive for the DNA of *Rickettsia* spp., *A. phagocytophilum*, *Ehrlichia* spp. and *Ca*. N. mikurensis, respectively. Although tick abundance was higher in natural habitats, the prevalence of the majority of pathogens was higher in urban forested areas.

**Conclusion:**

We conclude that: (i) zoonotic genetic variants of *A. phagocytophilum* are widely distributed in the Polish tick population, (ii) although the diversity of tick borne pathogens was higher in natural habitats, zoonotic species/strains were detected only in urban forests, (iii) and we provide the first description of *Ca.* N. mikurensis infections in ticks in Poland.

## Background

Pathogens that cause tick-borne diseases (TBDs), such as borreliosis or anaplasmosis, constitute a significant problem for the health of humans, companion animals and livestock worldwide. The majority of TBDs are classified as emerging infectious diseases because of the growing awareness of their importance, the availability of better diagnostic reagents and the consequent rising statistics for incidence in the human population [1,2]. In Europe, *Ixodes ricinus* is the most prevalent and widely distributed tick species and serves as the most important vector for several microbial pathogens [3-5]. However, tick abundance and the prevalence of transmissible pathogens in ticks varies locally depending on many factors, i. e. temperature, accessibility of suitable vertebrate hosts etc. [6-9]. The range of available hosts differs depending on the habitat characteristics (i.e. urban versus natural) and is likely to also influence the species/strain variability of pathogens vectored by ticks [10]. In urban habitats, humans, pets (mainly dogs), synantropic rodents (*Apodemus sylvaticus*) and birds probably play a significant role as tick hosts and the sources of TBPs. In natural forest habitats in Europe mainly deer (Cervidae), wild rodents (*Myodes glareolus, Apodemus flavicollis*) and birds are considered to be the most important hosts for *I. ricinus* and the major source of many TBDs [11-14].

In Poland, studies on the occurrence and diversity of *Rickettsia, Anaplasma, Ehrlichia* and ‘*Candidatus* Neoehrlichia’ in *I. ricinus* have been few and the subject has been relatively neglected when compared with other TBPs [15,16]. The best recognized is the occurrence of *A. phagocytophilum* in *I. ricinus* ticks, but little is known about the existence of co-infections and the species/strain diversity of other Rickettsiaceae and Anaplasmataceae species.

Tick-borne rickettsiosis is caused by intracellular bacteria belonging to the spotted fever group (SFG) of the Rickettsiaceae family. Since the early 1990s and the introduction and increasingly popular use of molecular methods (PCR, PCR-RFLP and sequencing), 17 new *Rickettsia* species have been added to the list of rickettsiae that are known to cause human diseases in different parts of the world [17]. Ticks are believed to act as vectors and reservoirs of the SFG group *Rickettsia* and interestingly, *Rickettsia* spp. are among the most common pathogens found in *Dermacentor reticulatus* and *I. ricinus* ticks in Europe [18]. However, reservoir hosts of *Rickettsia* are still not well defined and further investigations are required to elucidate the full range of potential host species.

The family Anaplasmataceae are gram-negative, intracellular bacteria, and include the genera *Anaplasma* and *Ehrlichia* as well as bacteria of the new candidate species ‘*Candidatus* Neoehrlichia mikurensis’ and ‘*Candidatus* Neoehrlichia lotoris’. It is already well established that *I. ricinus* ticks constitute competent vectors for *A. phagocytophilum,* an agent of human granulocytic anaplasmosis [19]. These bacteria are widespread over the territories of the USA, Europe and Asia and, apart from humans, can infect many mammalian species, i.e. dogs, cats, horses, sheep, rodents, birds and roe deer [14,20-22]. Molecular analyses have indicated that some strains/ genetic variants of *A. phagocytophilum* that are pathogenic for humans and domestic animals, circulate widely in nature in different hosts and display different vector tropisms and degrees of host pathogenicity [23-26].

Bacteria of a new candidate species ‘*Candidatus* Neoehrlichia mikurensis’ were discovered for the first time recently in small rodents and *I. ovatus* ticks in Japan [27]. It is believed that small rodents serve as reservoir hosts for *Ca.* N. mikurensis [28,29]*.* Moreover, human infections with these bacteria have been described recently in North and Central Europe as well as in China [30-33]. The occurrence of *Ca*. N. mikurensis DNA in *I. ricinus* ticks has been confirmed already in western and northern parts of Europe [34-36] and in *I. persulcatus* ticks in eastern Russia [20].

Among the five described *Ehrlichia* species, three cause infections in dogs and humans (*E. canis*, *E ewingii* and *E. chaffeensis*), *E. muris* is a rodent pathogen and *E. ruminantium* is an agent of heartwater disease in domestic ruminants [20]. The first case of *E. muris* infection was described originally in a wild-caught specimen of the mouse *Eothenomys kageus* in Japan [37] and since then single cases of *E. muris* infection have been detected in rodents [38] and *I. ricinus* ticks [39] in Europe. In Poland only one report has been published thus far and that was for the occurrence of *Ehrlichia* sp. in common voles, *Microtus arvalis*[40].

The key hypothesis driving our research was that tick abundance and hence the prevalence and the diversity of the TBPs that they carry should differ markedly between natural and urban forests where ticks abound, because of the contrasting ecology with either little or considerable human intervention, respectively, and the availability of different hosts for the ticks and hence reservoirs of varying competence for the pathogens. Our aims were therefore (1) to compare the densities of questing *I. ricinus* ticks (nymphs and adults) in forested urban and natural habitats; (2) to estimate the prevalence of *A. phagocytophilum, Rickettsia, Ehrlichia* and ‘*Ca.* Neoehrlichia spp.’ in *I. ricinus* ticks in these two different habitat types (urban and natural), and (3) to compare the species/strain diversity of tick-borne pathogens in two representative habitats in Central and Northeastern Poland.

## Methods

### Study sites

Five study sites in Central and North Eastern Poland were investigated in 2011. The two sites selected as examples of urban areas are located within the administrative borders of Warsaw city, near the city center (<8 km). Natural areas are situated in protected areas of national or landscape parks in rural settings at some distance from major conurbations.

### Urban areas

**Figure 1:**
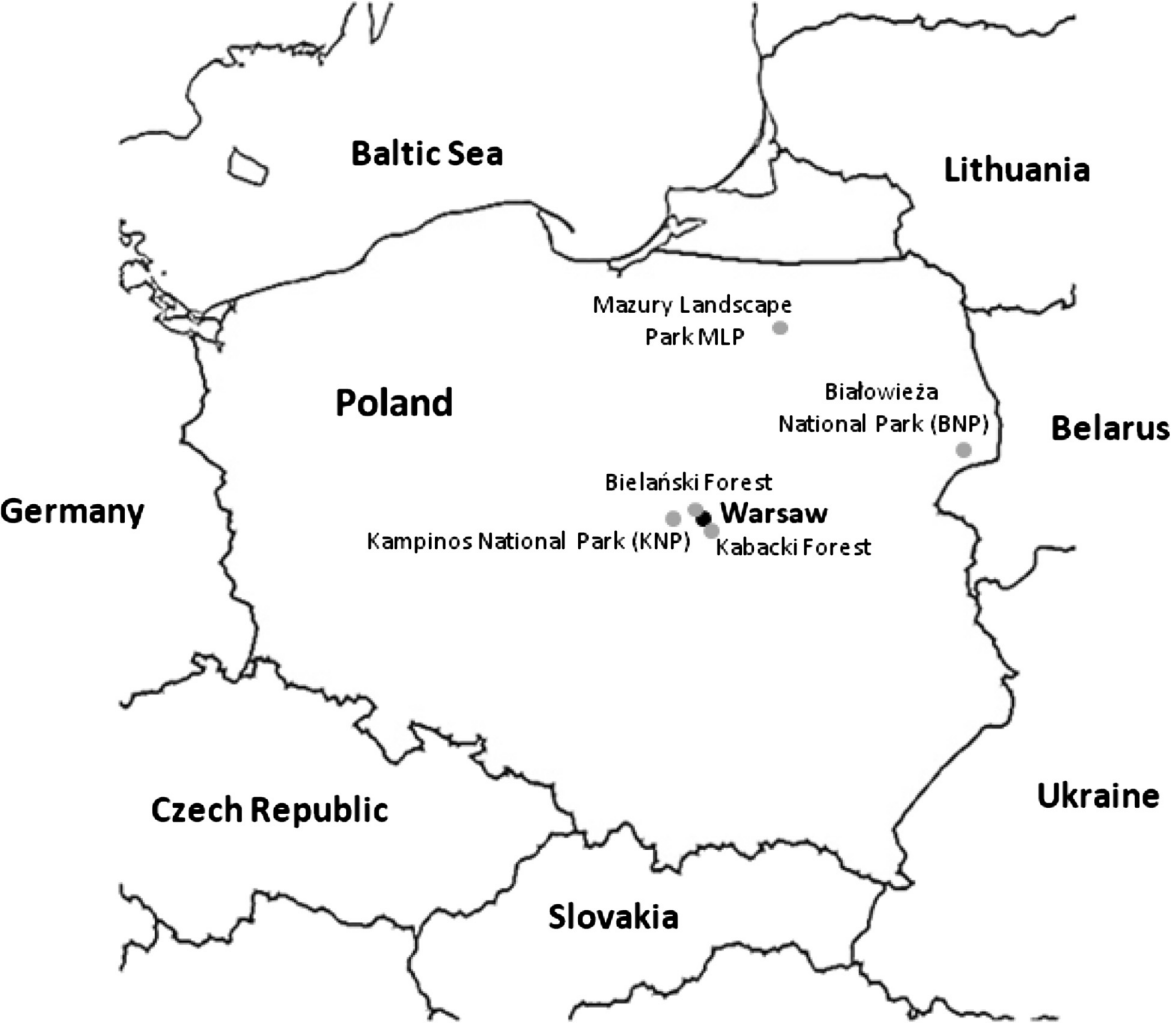
Map of the natural and urban areas in Central, North-eastern and East Poland where ticks were collected.

The Bielański [52°17’32”N, 20°57’36”E] and Kabacki Forests [52°6’58”N, 21°3’26”E] act as large city parks (130 ha and 903 ha, respectively), surrounded by dense urban and sub-urban settlements and are fragmented by paved roads and pedestrian/ cycle paths (Figure 1). The Kardynał Wyszyński University is situated in Bielański Forest. Access to urban forests is facilitated by main roads, the metro line and is within easy walking distance from nearby housing estates. These forests are affected by high anthropopression, being used daily as recreational areas by children, and intensively throughout the year by amateur cyclists, runners and country ski runners. The forests are also frequented by free-ranging feral dog populations and cats from the local housing estates. In Kabacki Forest, visitors are permitted to leave the paths to access facilities such as playgrounds for children, sports grounds, banks, approved camp fire/barbeque sites. In these urban parks, tick collection sites were selected near to the main park entrance (<1 km) and main roads. The fauna of Kabacki and Bielański forests is mainly limited to synantropic mammals such as rodents, urban foxes, hedgehogs, martens, badgers and a variety of species of birds. Roe deer, hare and wild boar may also occasionally venture into these locations. Elk migrating from the Kampinoski National Park may be occasionally encountered in Bielański Forest.

### Natural areas

Three of the five study sites (Kampinoski National Park, **KNP** [52°18’21”N, 20°36’32”E], Białowieża National Park, **BNP** [52°46’20”N, 23°50’60”E], Mazury Landscape Park, **MLP** [53°48’25”N, 21°38’36”E]) are situated in protected areas of national or landscape parks and were considered as ‘natural’ areas, in Central (KNP) and Northeastern Poland (BNP, MLP) (Figure 1). Białowieża National Park (62 500 ha) has a global reputation as a model natural habitat, representative of the ancestral European primeval deciduous forests (World Heritage List of UNESCO since 1972). It is known also for its populations of game species, including roe and red deer and wild boar, and in particular for its free-living population of European bison (*Bison bonasus*). Among other mammalian species regularly encountered in the forest are wolves, elks, lynx and beavers.

Both Kampinoski National Park and Mazury Landscape Park comprise a mixture of natural and managed forests, some of the latter having been managed for over 120 years in the case of MLP. Managed forests consist of pine trees (*Pinus sylvestris*) with the addition of deciduous tree species (birch, alder, elm and oak). MLP is well known for its combination of impressive forests and numerous (>1000) natural lakes. These forests are not fragmented, but are surrounded by agricultural areas, with minimal numbers of human settlements and are inhabited by a range of wild game and protected species indigenous to the region. As with BNP these include: roe and red deer, wild boar, elk, lynx, wolf, beaver and many other species of small and medium size mammals (i.e fox, badger, hare, marten) and birds. Human activity in the forests is very limited and seasonal; hunters, local people collecting berries or mushrooms, tourists (mainly cyclers), forestry workers. It is mandatory to keep dogs on the leash in the national parks in Poland, otherwise entry is prohibited by law and contravention is enforced by fines.

As far as we are aware there are no quantitative census data through which anthropopression can be reliably compared between our urban and natural study sites, but from our personal experience of having visited our study sites regularly we estimate the number and density of visitors to be 1000-10000 higher in the urban parks compared to the natural forests. Similarly, the predator pressure from domestic and feral dogs and cats is undoubtedly much higher in the two urban forests, as opposed to the natural predator-prey relationships that exist in natural forests.

### *Ixodes ricinus* ticks

Questing ticks, only nymphs and adults, were collected from vegetation by blanket dragging (white blanket 1.5×0.75 m) in central and north-eastern regions of Poland in May, July and September 2011. Ticks were collected twice daily (about 9 a.m. and 4-5 p.m.), for a minimum of four consecutive days in each month. In each collection site, tick abundance was measured by conventional flagging in 300-500 m^2^ plots and then extrapolated to a standard 100 m^2^. The species, developmental stage of individual ticks, as well as the sex of adults were recorded, and the nymphs from a single drag during flagging were pooled in groups of 10 prior to DNA extraction. In the case of positive PCR results for nymphs, it was assumed that at least one nymph was infected (minimal infection rate; MIR). Genomic DNA was extracted from homogenized tick specimens using the DNAeasy Blood and Tissue Kit (Qiagen, Crawley, UK ) and stored at –20°C.

### PCR analysis

Detection and genotyping of *Rickettsia*, *A. phagocytophilum, Ehrlichia* spp. and *Ca.* Neoehrlichia spp. were performed by amplification and sequencing of different loci: the *groESL* heat shock operon and *16S rRNA* gene for the Anaplasmataceae family and *gltA* for the Rickettsiaceae family. The primers and thermal profiles used in this study have been described previously [19,41,42]. Reactions were performed in a final volume of 20 μl and contained 0.33 mM dNTPs (Eurobio, Lille, France), 2 mM MgCl_2_, 1× PCR buffer, 1 U Taq polymerase (Fermentas), 1 μM of each primer and 5 μl of the extracted DNA sample. Negative controls were performed in the absence of template DNA. To check whether amplifiable DNA had been extracted, we used PCR reactions employing tick-specific primers [43]. Only positive samples were chosen for further analysis. Amplicons were visualized with Midori Green stain (Nippon Genetics Europe GmbH) following electrophoresis in 2% agarose gels. Amplicons were purified using the Axygen Clean-up purification kit (Axygen, USA) and sequenced by a private company (Genomed S.A., Poland) in both directions.

### Phylogenetic analysis

DNA sequence alignments and phylogenetic analysis were conducted using MEGA version 5.0 [44]. After testing the data for the best substitution model, phylogenetic trees were obtained using Maximum Likelihood as the tree construction method and Hasegawa-Kishino-Yano parameter algorithm as a distance method. For comparison, sequences of *Rickettsia, A. phagocytophilum, Ehrlichia* and *Ca.* Neoehrlichia species/strains obtained from GenBank (http://www.ncbi.nlm.nih.gov) were implemented in the sequence alignment. The stability of inferred phylogenies was assessed by bootstrap analysis of 1000 randomly generated sample trees.

### Statistical analysis

Data are expressed as arithmetic means ± 95% confidence limits per 100 m^2^ of blanket dragging. Tick abundance was analyzed using multifactorial general linear models (GLM) with normal error structures in SPSS v.20 for Windows. We fitted quality of habitat (HABITAT, 2 levels- urban [Bielański + Kabacki Forests] and natural [KNP + BNP + MLP]), SITE (5 levels: KNP, BNP, MLP, Bielański Forest, Kabacki Forest) and tick STAGE of development (3 levels: nymphs, females and males) as factors. Differences in *Rickettsia, A. phagocytophilum, Ehrlichia* and *Ca.* Neoehrlichia prevalence in ticks between study sites were analyzed using the χ^2^ test in Instat or Fisher Exact Test when applicable.

### New nucleotide sequences

New nucleotide sequences have been deposited in GenBank with the accession numbers KF312351-KF321354 for *16S rRNA* of *A. phagocytophilum*, KF312355-KF321361 for *groESL* of *A. phagocytophilum*, KF312362 for *groESL* of *E. muris* and KF312363 for *groESL* of *Ca*. N. mikurensis.

## Results

### Abundance of *I. ricinus* ticks

Overall a total of 1589 ticks were collected and of these 72% (1147) were nymphs and 28% were adults (241 males and 201 females) (Table 1). The abundance of questing *I. ricinus* (nymphs and adults) varied significantly between study sites (GLM, main effect of SITE; *F*_4_,_140_ = 4.80, *P* = 0.001). The highest tick density per 100 m^2^ was recorded in Białowieża National Park (Table 1). Average tick densities were similar in Bielański Forest and MLP. The overall abundance of *I. ricinus* was lowest in KNP and Kabacki Forest (Table 1). The abundance of nymphs was highest in Bielański Forest and BNP (GLM, main effect of SITE; *F*_4,140_ = 3.90, *P* = 0.002, Table 1). Female and male abundances were relatively low (0.6-2.8 ticks per 100 m^2^) and no significant differences were observed between sampling areas. The abundance of *I. ricinus* ticks was almost twice as high in natural as in urban areas (GLM, main effect of HABITAT; *F*_1,140_ = 5.80, *P* = 0.017) (Table 1). Because the difference in the average tick densities per 100 m^2^ between the two types of forest was significant, the sampling areas were divided into two groups with: (1) low tick abundance (5.7 [2.7-8.7]; Kabacki Forest and KNP), and (2) high tick abundance (14.5 [12.2-16.7]; Bielański Forest, BNP, MLP) (GLM, main effect of HABITAT; *F*_1,140_ = 21.2, *P* < 0.001) (Table 1). No statistical differences in tick abundance between study months were observed.

**Table 1:** **Abundance of *I. ricinus *** **ticks in forested urban and natural regions of Poland**

**Collection site**	**Quality of habitat**	**Abundance of *Ixodes ricinus *** **ticks (per 100 m**^**2 **^**[95% CL])**												**Total, all months**				
		**May**				**July**				**September**								
		**Nymphs**	**Females**	**Males**	**Total**	**Nymphs**	**Females**	**Males**	**Total**	**Nymphs**	**Females**	**Males**	**Total**	**Nymphs**	**Females**	**Males**	**Total**	
Bielański Forest	urban	8.3	0.9	1.2	10.5	17.5	4.0	0.5	22.0	9.5	2.0	1.5	13.0	11.8	2.3	1.1	15.2*	8.8
		[5,3-11,3]	[0,1-1,8]	[0,2-2,3]	[6,8-14,1]	[5,4-29,6]	[0,4-7,6]	[0,0-4,9]	[7,2-36,8]	[0,0-21,6]	[0,0-5,6]	[0,0-5,9]	[0,0-27,8]	[6.0-17.6]	[0.6-4.0]	[0.0-3.2]	[8.1-22.2]	[5.9-11.6]
Kabacki Forest		3.7	0.2	0.3	4.2	6.0	1.5	1.0	8.5	9.5	0.0	1.5	11.0	6.4	0.6	0.9	7.9	
		[0,0-7,5]	[0,0-1,3]	[0,0-1,6]	[0,0-8,8]	[0,0-18,1]	[0,0-5,1]	[0,0-5,4]	[0,0-23,3]	[0,0-21,6]	[0,0-3,6]	[0,0-5,9]	[0,0-25,8]	[0.6-12.2]	[0.0-2.3]	[0.0-3.0]	[0.8-15.1]	
BNP	natural	11.5	3.4	3.9	18.8	21.3	2.3	2.7	26.3	8.2	2.2	1.7	12.1	13.7	2.6	2.8	19.1*	13.4
		[7,6-15,5]	[2,2-4,5]	[2,5-5,4]	[14,0-23,6]	[14,3-28,3]	[0,3-4,4]	[0,2-5,2]	[17,8-34,9]	[2,5-13,9]	[0,5-3,9]	[0,0-3,7]	[5,1-19,1]	[10.4-17.0]	[1.7-3.6]	[1.6-3.9]	[15.1-23.1]	[10.9-16.0]
KNP		1.7	2.1	2.9	6,8	2.3	0.8	1.3	4.5	6.0	0.3	1.0	7.3	3.4	1.1	1.8	6.2	
		[0,0-6,2]	[0,8-3,4]	[1,3-4,5]	[1,4-12,2]	[0,0-9,3]	[0,0-2,9]	[0,0-3,8]	[0,0-13,0]	[0,0-14,6]	[0,0-2,8]	[0,0-4,1]	[0,0-17,7]	[0.0-7.3]	[0.0-2.2]	[0.3-3.2]	[1.3-11.0]	
MLP		13.3	0.3	0.5	14,1	3.0	0.0	5.0	8.0	10.0	2.0	2.4	14.4	8.8	0.8	2.6	12.2*	
		[8,6-18,1]	[0,0-1,7]	[0,0-2,2]	[8,3-19,9]	[0,0-20,1]	[0,0-5,0]	[0,0-11,2]	[0,0-28,9]	[2,3-17,7]	[0,0-4,3]	[0,0-5,2]	[5,0-23,8]	[2.3-15.2]	[0.0-2.7]	[0.3-4,9]	[4.2-20.0]	
Total, all sites		7.7	1.4	1.7	10.9	10.0	1.7	2.1	13.9	8.6	1.3	1.6	11.6	8.8	1,5	1.8	12.1 [9.3-14.9]	
		[5.9-9.5]	[0.9-1.9]	[1.1-2.4]	[8.7-13.1]	[4.8-15.2]	[0.2-3.3]	[0.2-4.0]	[7.5-20.3]	[4,4-12.9]	[0.0-2.6]	[0.1-3.2]	[6.3-16.8]	[6.5-11.1]	[0.8-2.2]	[1.0-2.7]		

CL- 95% confidence limits.

^*^classified as high-tick-density forests.

N- nymphs, F- females, M-males.

### Prevalence of pathogens in ticks

Of the 1325 adults and nymphs, a total of 6.2% (*n* = 82) were infected with at least one pathogen. As expected, the prevalence of pathogens in ticks was significantly higher in adults (10.0%; 42/421) than the MIR in nymphs (4.4%; 40/904) (*χ*^2^ = 14.31, *df* = 1, *P* < 0.0001). Prevalence of pathogens in ticks differed between urban and natural habitats (Table 2). Overall prevalence of TBPs was almost three times higher in urban compared with natural forests (χ^2^ = 22.97, df = 1, P < 0.0001).

**Table 2:** **The prevalence of tick-borne pathogens in *I. ricinus *** **collected in forested urban and natural areas of Poland**

**Site**	**Quality of habitat**	**Overall TBD prevalence%**	**Prevalence% (no. infected/no. tested)**							
			***Rickettsia *** **sp.**		***A. phagocytophilum***		***Ca. *** **Neoehrlichia sp.**		***Ehrlichia *** **sp.**	
Bielański Forest^*^	urban	11.6 (42/405)	4.5 (9/201)	7.7 (31/405)	6.0 (12/201)	3.0 (12/405)	0.0 (0/201)	0.2 (1/405)	0.0 (0/201)	0.7 (3/405)
Kabacki Forest			10.8 (22/204)		0.0 (0/204)		0.5 (1/204)		1.5 (3/204)	
Białowieża National Park^*^	natural	4.4 (40/920)	1.6 (10/625)	2.9 (27/920)	1.3 (8/625)	1.1(10/920)	0.0 (0/625)	0.2 (2/920)	0.0 (0/625)	0.1 (1/920)
Kampinos National Park			3.8 (6/156)		1.3 (2/156)		0.0 (0/156)		0.0 (0/156)	
Mazury Lake District^*^			7.9 (11/139)		0.0 (0/139)		1.4 (2/139)		0.7 (1/139)	

^*^classified as high-tick-density forests.

### *Rickettsia* spp. infections

This was the predominant pathogen in our study, 4.4% (58/1325) of the tested ticks being recorded as positive for *Rickettsia* spp. The prevalence of infected ticks was about 2.5 times higher in urban (7.7%) than in natural sites (2.9%) (*χ*^2^ = 7.77, *df* = 1, *P* = 0.0053). The DNA of *Rickettsia* spp. was detected in 3.7% nymphs (MIR) (33/904) and in 5.9% adults (25/421) (NS). Prevalence of *Rickettsia* was significantly higher in low-tick-density forests (7.8% in KNP + Kabacki Forest) than in high-tick-density forests (3.1% in BNP + MLP + Bielański Forest) (*χ*^2^ = 12.60, *df* = 1, *P* < 0.001).

Sequence analysis of the 770 bp fragment of the *gltA* gene of 40 isolates (20 of each habitat type, urban and natural) showed the presence of two different *Rickettsia* species. The most prevalent was *R. helvetica* (38/40, 95%), closely related (99.9% homology) to the *R. helvetica* isolates [GenBank: EU359285] originally obtained from *Ixodes* ticks in Switzerland and differed from them by only one nucleotide (G → T) at position 713. *Rickettsia helvetica* isolates were found in all study sites. Two of 40 isolates (5%) were identified as *R. monacensis*. These isolates showed 99.7% similarity (nucleotide substitutions at positions 476 [T → C] and 713 [G → C]) to *R. monacensis* IrR/Munich strain [GenBank: DQ100163] derived from *I. ricinus* in Germany and were identical with *R. monacensis* isolated from human patients in Spain [GenBank: DQ517498] and Korea [GenBank: FJ009429]. The two *R. monacensis* isolates both originated from an urban forest (Kabacki Forest).

### *Anaplasma phagocytophilum* infections

The overall prevalence of *A. phagocytophilum* in *I. ricinus* was 1.7% (22/1325). Prevalence was significantly higher in urban than in natural sites (3% versus 1.1%) (Table 2, *χ*^2^ = 4.98, *df* = 1, *P* = 0.026). No positive ticks were found in Kabacki Forest and Mazury Landscape Park forest. The MIR in nymphs was significantly lower (0.6%; 5/904) than the prevalence in adults (4.0%; 17/421) (*χ*^2^ = 18.26, *df* = 1, *P* < 0.0001), and prevalence did not differ statistically between ticks from low- and high-tick-density forests (Table 2).

**Figure 2:**
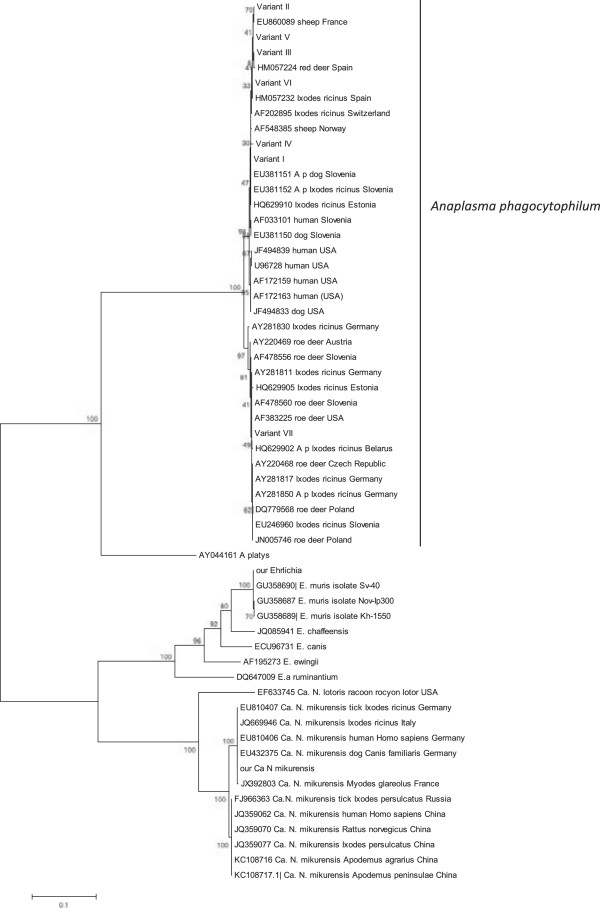
**Phylogenetic tree of the *A. phagocytophilum, Ehrlichia i Ca. *Neoehrlichia isolates studied in the current work and chosen isolates from GenBank based on the fragment of the *groESL *heat shock operon.** Numbers at the nodes of the tree indicate bootstrap values (1000 replicates).

The 540 bp fragment of the *16S rRNA* gene and the 1200 bp fragment of the *groESL* heat shock operon were further analyzed in 18 isolates. The nucleotide identity/similarity of the sequenced *16S rDNA* fragments was very high (99.6-100%). Eight of 18 sequences from natural areas (Table 3; KNP + BNP) were identical, representing genetic variant I, which is considered to be zoonotic (Figure 2). A further eight isolates could be distinguished on the basis of substitution at position 376 (G → A) in the variable region near the 5’ end of the *16S rRNA* gene, representing genetic variant II (Table 3). Variant III was represented by one isolate and differed by two nucleotides at positions 199 (G → T) and 376 (G → A). Variant IV included one isolate that differed by two nucleotides at position 376 (G → A) and 539 (G → T). Isolates belonging to variant I were found in ticks collected from natural areas. Isolates of variants II, III and IV were found only in ticks from one urban forest- Bielański Forest (Table 3).

**Table 3:** **Polymorphism in the fragment of *16S rRNA *** **gene in *A. phagocytophilum *** **isolates from ticks and human pathogenic strains (sequences published in GenBank)**

**Strain/genetic variant**		**Nucleotide positions 5’ → 3’**^**a**^				**Quality of habitat/country**	**No. of isolates (reference no. of samples)/site of study**	**GenBank acc. number**	**Host/vector found in other studies (only 100% homology; nucleotide sequences deposited in GenBank)**
		**199**	**328**	**376**	**539**				
This study	I	G	A	G	G	natural/ Poland	**2** (6,153)/KNP	KF312352	bank vole (*Myodes glareolus*, KC740432); dog (KF985242); elk (*Alces alces*, KC800983); roe deer (*Capreolus capreolus*, JQ965530); cotton rat (*Sigmodon hispidus*, JQ063025); rat (*Rattus norvegicus*, KC470064); reindeer (*Rangifer tarandus*, JX841254); hedgehog (*Erinaceus europaeus*, JN571163); *I. ricinus* (KF481930), *I. trianguliceps* (KF481934)
							**6** (39, 54, 73, 85, 90, 283)/BNP		
	II	G	A	**A**	G	urban/Poland	8 (29, 37, 38, 47, 62, 67, 71, 73)/Bielański Forest	KF312353	dog (EF668225); cat (HM138366); *I. ricinus* (JX909354); *D. reticulatus* (KF381413)
	III	**T**	A	**A**	G		1 (57)/Bielański Forest	KF312351	n. m.^b^
	IV	G	A	**A**	**T**		1 (64)/Bielański Forest	KF312354	n. m.^b^
*A. phagocytophilum* strains pathogenic for human		G	A	G	G	Poland		KF111754	
		G	A	G	G	Slovenia		GU236658	
		G	A	G	-	Italy		DQ029028	
		G	A	G	G	USA		GU236664	
		G	A	G	G	USA		NR_074113	

^a^The number corresponds to the positions of nucleotide substitutions relative to the sequence of the complete *16S rRNA* gene of *A. phagocytophilum* strain HZ (NR_074113). Base substitutions are shown in bold.

^b^No match has been found in GenBank.

The nucleotide sequences of isolates from groups I and II were identical to the sequences obtained from rodents, dogs, cats, roe deer, hedgehog and ticks (Table 3). Additionally, the *16S rRNA* sequence of variant I was identical with the human pathogenic strains isolated in Slovenia, Italy and the USA (Table 3). The nucleotide sequences of isolates from groups III and IV did not show 100% identity to the known sequences of *16S rRNA* of *A. phagocytophilum* deposited in GenBank.

The partial *groESL* fragments (1200 bp) were also sequenced for all positive samples (n = 18) since the *16S rRNA* gene is too conserved for analysis of genetic heterogeneity. The level of homology between isolates was also high (98.0-100%). Sequence analysis allowed identification of seven different genetic variants (I-VII) that differed by 2 to 13 nucleotides (Table 4). Genetic variants I and II included 10 isolates from ticks collected only in Bielański Forest, an urban site (Table 4). Variants III-VII were composed of 1 or 2 sequences found only in ticks from natural forested areas (BNP, KNP). Analysis of these *groESL* fragments allowed the differentiation of 4 variants from BNP, all grouped in variant I based on the *16S rRNA* gene fragment (Table 3).

**Table 4:** **Polymorphism in the fragment of the *groESL *** **heat shock operon in *A. phagocytophilum *** **isolates from ticks and human pathogenic strains (sequences published in GenBank)**

**Strain/genetic variant**		**Nucleotide positions 5’ → 3’**^**a**^																													**Quality of habitat/country**	**No. of isolates (reference no. of samples)/site of study**	**GenBank acc. number**	**Host/vector found in other studies (only 100% homology; nucleotide sequences deposited in GenBank)**
		**167**	**398**	**401**	**431**	**476**	**486**	**497**	**511**	**561**	**776**	**821**	**836**	**837**	**840**	**845**	**857**	**872**	**890**	**923**	**956**	**965**	**980**	**992**	**1049**	**1085**	**1088**	**1112**	**1229**	**1244**				
This study	I	G	A	A	G	C	G	T	C	**A**	C	C	A	C	T	A	C	C	G	C	C	G	T	T	C	A	C	C	**A**	**T**	urban/Poland	9 (29, 37, 38, 47, 57, 62, 67, 71, 73)/ Bielański Forest	KF312357	dog (EU381151); *I. ricinus* (EU381152)
	II	G	A	A	G	C	G	**C**	C	**A**	C	C	A	C	T	A	C	C	**T**	C	**T**	G	T	T	**T**	A	C	C	G	C		1 (64)/ Bielański Forest	KF312360	red deer (*Cervus elaphus*, HM057225); I. ricinus (KF383239)
	III	G	A	A	**A**	C	G	T	C	**A**	C	C	A	C	T	A	C	C	**T**	C	**T**	G	T	T	**T**	A	C	C	G	C	natural/Poland	2(85, 90)/BNP	KF312358	*I. ricinus* (AY281831)
	IV	G	A	A	G	**A**	**A**	T	**G**	**A**	C	C	A	C	T	A	C	C	G	C	C	G	T	T	C	A	C	C	**A**	**T**		2(39, 54)/BNP	KF312355	n. m. ^b^
	V	G	A	A	G	C	G	**C**	C	**A**	C	C	A	C	T	A	C	C	G	C	**T**	G	T	T	**T**	A	C	C	**A**	**T**		1(73)/BNP	KF312359	n. m ^b^
	VI	**A**	A	A	G	C	G	**C**	C	**A**	C	C	A	C	T	A	C	C	G	C	**T**	G	T	T	C	A	C	C	G	C		1(283)/BNP	KF312361	n.m ^b^
	VII	G	**G**	A	G	C	G	T	C	**A**	C	**A**	**G**	**G**	**G**	**T**	**T**	**T**	G	**G**	C	**A**	**A**	**C**	**T**	**G**	C	**T**	G	C		2(6, 153)/KNP	KF312356	roe deer (*Capreolus capreolus*, AF478560); *I. ricinus* (EU552914)
*A. phagocytophilum* strains pathogenic for human		G	A	**G**	G	C	G	T	C	G	C	C	A	A	T	A	C	C	G	C	C	**A**	T	T	C	**G**	**A**	**T**	G	C	USA		U96728	
		G	A	A	G	C	G	T	C	G	C	C	A	A	T	A	C	C	G	C	C	**A**	T	T	C	**G**	**A**	**T**	G	C	USA		AF172159	
		**A**	A	A	G	C	G	T	C	G	**T**	C	A	A	T	A	C	C	G	C	C	G	T	T	C	A	C	C	**A**	**T**	Slovenia		AF033101	
		G	A	**G**	G	C	G	T	C	G	C	C	A	A	T	A	C	C	G	C	C	**A**	T	T	C	**G**	**A**	**T**	G	C	USA		JF494839	

^a^The number corresponds to the positions of nucleotide substitutions relative to the sequence of the *groESL* heat shock operon of the human pathogenic *A. phagocytophilum* strain (U96728). Base substitutions are shown in bold.

^b^No match has been found in GenBank.

Scrutiny of the phylogenetic tree, based on the partial *groESL* operon sequences, showed that two isolates from variant VII were identical to other European isolates, mainly from *I. ricinus* ticks and roe deer (Figure 2, Table 4). Isolates belonging to variant I-III clustered with *A. phagocytophilum* pathogenic for human and domestic animals in Europe, as well as in North America, and differed from variant VII by 13 nucleotides (Table 4). Thus, these 6 variants (I-VI) are considered to represent zoonotic genotypes. The nucleotide sequences of isolates from groups IV-VI did not show 100% identity to the known sequences of *groESL* operon sequences of *A. phagocytophilum* deposited in GenBank.

### *Ehrlichia* spp. and *Candidatus* Neoehrlichia spp. infections

The DNA of *Ehrlichia* spp*.* and that of *Ca.* Neoehrlichia spp*.* was found in 0.3% (4/1325; only in nymph pools) and 0.2% (3/1325; two males and 1 nymph pool) of the sampled ticks, respectively. Three ticks positive for *Ehrlichia* DNA were collected in an urban area (Kabacki Forest) and one in MLP (natural site) (Table 2). The presence of *Ca*. Neoehrlichia spp. was confirmed in nymphs from Kabacki Forest (urban area) and in 2 males from MLP (natural site) (Table 2).

Molecular analysis of the 540 bp fragment of the *16S rRNA* gene of *Ehrlichia* (n = 4) and *Ca*. Neoehrlichia (n = 3) isolates showed that all sequences were identical and differentiation between these genera was possible only on the basis of the partial *groESL* fragment (1200 bp). The four *Ehrlichia* isolates were identical [GenBank: KF312362] and showed 82.3% *groESl* sequence homology with *Ca*. Neoehrlichia and 100% sequence homology with *E. muris* originally obtained from rodents [GenBank: GU358690] and *I. persulcatus* [GenBank: GU358686] in Russia. All *Ca*. Neoehrlichia isolates were also identical [GenBank: KF312363] and showed 100% sequence homology with *Ca*. N. mikurensis from *I. ricinus* ticks [GenBank: EU810407] and humans [GenBank: EU810406] in Germany. Our isolates clustered also with other *Ca*. N. mikurensis pathogenic for humans [GenBank: HM045824] and dogs [GenBank: EU432375] in Europe (Figure 2).

### Co-infections in *I. ricinus* ticks

4.8% (2/42) of adult ticks identified as carrying pathogens yielded positive results for two pathogens and none were infected with three or more. *Rickettsia helvetica* and *A. phagocytophilum* were involved in both co-infections. Both ticks co-infected with these pathogens were collected in an urban area (Bielański Forest). Analysis of co-infections in nymphs was not justified because DNA was extracted from pooled samples of ticks.

## Discussion

The results of our study, focusing on a comparison of the abundance of *I. ricinus* ticks and prevalence of TBPs in ticks sampled in forested urban and natural habitats, revealed significant and interesting differences between these ecologically contrasting sites. Although tick abundance was higher in natural habitats, the prevalence of the majority of TBPs (*A. phagocytophilum, Rickettsia* spp.*, E. muris*) was higher in urban areas. Interestingly, while the diversity of *A. phagocytophilum* was higher in natural habitats, only the zoonotic species/ strains (i.e. *R. monacensis, A. phagocythophilum* variants I-II [*groESL* operon sequences]) were detected in urban forests. This finding likely reflects the availability of different reservoir hosts and points to a higher risk of TBDs for humans venturing into urban forests compared with forests located in natural sites. We also report here for the first time the detection of *Candidatus* Neoehrlichia mikurensis in questing *Ixodes ricinus* ticks in Poland.

As in many previous studies on ticks [45,46], in our study also the abundance of questing *I. ricinus* varied significantly between study sites. The highest density was recorded in natural forested habitats, i.e. in the Białowieża National Park and the Mazury Landscape Park both of which correspond to the ‘medium tick abundance’ category of forests (11-40 ticks per 100 m^2^[47]). A similar high abundance was observed in Bielański Forest in Warsaw, which is a part of an ancient deciduous primeval forest and the soil humidity and plant coverage are very similar to those encountered in Białowieża Primeval Forest (BNP). Deciduous humid forests are known to constitute preferable habitats for *I. ricinus* ticks [48,49]. The overall abundance of *I. ricinus* was lowest in Kampinos National Park and Kabacki Forest, where pine *Pinus silvestris* are the main tree species because of the poorer sandy soil conditions that are less suitable for deciduous tree species (mainly low-humid sandy areas; ‘low tick abundance’, 3-10 ticks per 100 m^2^[47]).

Despite lower tick abundance, the prevalence of three of four pathogens detected in this study was at least twice as high in ticks from urban forests, a finding corresponding to the so-called ‘dilution effect’ of Estrada-Peña *et al.*[50]. The reasons underlying this difference and its consequences are of particular relevance for public health concerns. For an explanation of this phenomenon we looked at the genetic diversity of the most common pathogens. Although the diversity of *A. phagocytophilum* was higher in natural habitats, only zoonotic species/ strains (i.e. *R. monacensis, A. phagocythophilum* variants I-II [*groESL* operon sequences]) were detected in urban forests. This finding likely reflects the availability of different reservoir hosts for the pathogen and is consistent with the idea that in urban areas humans, companion animals (mainly dogs) as well as synanthropic rodents, foxes and hedgehogs constitute suitable hosts for ticks and act as reservoir hosts for the parasites. *Rickettsia monacensis*, which is pathogenic for humans, was found only in ticks from Kabacki Forest, supporting our hypothesis and pointing to a new health risk in this recreational area of Poland’s capital city, Warsaw. The greatest genetic variability of *A. phagocytophilum* (5 variants) was found in ticks from Białowieża and Kampinos National Parks (including also the non-zoonotic variant VII [*groESL* operon sequences]) probably reflecting the wide range of natural *I. ricinus* hosts that live in these forests and can act as reservoir hosts for *A. phagocytophilum* (i.e. Cervidae, Carnivores). In this context it is pertinent also that in our earlier studies on roe deer, the dominance of non-zoonotic over zoonotic *A. phagocytophilum* strains and other TBPs (i.e. *Babesia, Bartonella*) was noted [14]. Thus, although tick density and TBP diversity in ticks in urban areas appeared to be lower than in the natural habitats and to depend on a narrower tick host range, the risk for humans may be higher, as humans ‘share’ many TBPs with dogs, livestock and rodents, as a result of many years of co-existence.

Previous molecular studies have shown relatively high prevalence of tick-borne pathogens in *I. ricinus* from different parts of Europe. The most common infection in the current study was *Rickettsia* spp. with a prevalence of 4.4%, and this is comparable to prevalence rates reported from other countries in Europe: 5% in Luxembourg [51], 14-17% in Germany [6], 1.4% in France [52], 11% in Slovakia [38] and 9% in Poland [53]. In contrast to the results obtained by Overzier *et al.*[10] and Venclikova *et al*. [54], a significantly higher prevalence of *Rickettsia* was found in urban than in natural areas (7.7 vs. 2.9%). On the basis of the *gltA* gene we have identified two different *Rickettsia* species. Isolation of *R. helvetica* in our study confirmed the high prevalence and widespread nature of this species in *I. ricinus* in Europe. Since 1999, several *R. helvetica* seropositive patients with relatively mild, self-limited illnesses associated with flu-like symptoms have been reported, so the pathogenicity of *R. helveti*ca needs further investigation. The nucleotide sequences of the *gltA* fragment gene showed that our *R. monacensis* isolates were identical with the *R. monacensis* strain Rp-Sp1 that is known to be pathogenic for humans. *R. monacensis* is an etiological agent of human rickettsiosis in Spain [55] and the DNA of these bacteria has been detected in an asymptomatic patient in Croatia [56]. The presence of these bacteria in *I. ricinus* ticks has also been confirmed in other European countries including Germany and Romania [57,58]. In Poland, *R. monacensis* has been detected recently in Northwestern Poland (in Szczecin, near Poland’s western border with Germany), where this species has never been recorded previously in ticks [53].

*A. phagocytophilum* has been detected in *I. ricinus* ticks throughout Europe and the prevalence rate has been reported to vary from 1 to 5% in Poland, Switzerland, Russia and Belarus [59-61], however, locally it may exceed 24% (Italy [62]). As in our results with *Rickettsia* and the data of Venclikova *et al*. [54], we found a significantly higher prevalence of *A. phagocytophilum* in urban compared with natural areas (3.0 vs. 1.1%). In agreement with data published previously [26], sequence analysis of the 1200-bp fragment of *groESL* in the current work revealed two distinct genetic lineages of *A. phagocytophilum*: (1) genetic variants detected in humans, ticks, dogs, horses, sheep and red deer from Europe and USA which are believed to be pathogenic; and (2) genetic variants isolated from ticks and roe deer in Europe that are probably non-zoonotic strains [61,63]. In our study, molecular analysis of the *16S rRNA* fragment gene showed that the nucleotide sequences of eight *A. phagocytophilum* isolates belonging to genetic variant I were identical with *A. phagocytophilum* strains known to be pathogenic for humans (Table 3). However, on the basis of molecular and phylogenetic analysis of the *groESL* fragment, two isolates from variant I (no. 6 and 153), were assigned to the non-zoonotic variant VII. Therefore, our results emphasize that analysis of the nucleotide sequences of several genes is necessary, indeed essential, for a detailed delineation of different *A. phagocytophilum* strains [20].

This study has reported for the first time the presence of ‘*Ca*. N. mikurensis’ in questing *I. ricinus* from Poland. The prevalence rates of ‘*Ca*. N. mikurensis’ and *E. muris* in our study were low and differed from those in reports from Slovakia (*E. muris* 3% [39]), Switzerland (*Ca*. N. mikurensis 6% [64]) and Germany (*Ca*. N. mikurensis 6% [35]). In our study, the 540-bp fragment of the *16S rRNA* gene was highly conserved and differentiation of the two genera was possible only on the basis of the *groESL* operon fragment. Although *E. muris* is mainly a murine pathogen, ‘*Ca*. N. mikurensis’ is now considered to be an emerging tick-borne pathogen of veterinary and medical importance [65]. Isolates from this study were identical with ‘*Ca*. N. mikurensis’ detected in blood samples of patients with severe febrile illnesses and with the strain isolated from *I. ricinus* from Germany [32]. Our results confirm the presence of ‘*Ca*. N. mikurensis’ in questing *I. ricinus* from Poland and point to a new risk of infection with a recently discovered TBP for animals and humans.

Interestingly, both ticks in which two genera of TBPs were detected (*R. helvetica/ A. phagocytophilum*) were collected in Bielański Forests from the area of Warsaw. Thus, the risk of acquiring double infection from the infected ticks appears to be higher in urban areas. However, because the number of detected co-infections was so low, on the basis of the current results it is not possible to conclude whether these concurrently infected ticks can be considered as constituting an additional health risk for people visiting the city forests.

## Conclusions

There were significant differences in tick abundance between natural and urban forested habitats. Although tick abundance was higher in natural habitats, the prevalence of the majority of TBPs was higher in urban areas. This finding, together with the detection of zoonotic species/strains of Rickettsiaceae and Anaplasmataceae indicates a high risk of TBDs in city forests. The new pathogen- *Candidatus* Neoehrlichia mikurensis- was detected for the first time in questing *I. ricinus* ticks in Poland, also in urban areas.

## Competing interests

The authors declare that they have no competing interests.

## Authors’ contributions

Designed the study, acquired the funding: ES, RWF; collected and identified ticks: MK, GK, ES; processed samples: MK, RWF; performed PCR: MK, RWF; analyzed sequences: RWF; analyzed the data: RWF, AB, MK, JMB; wrote the paper: RWF, AB, JMB, ES. All authors read and approved the final version of the manuscript.

## References

[B1] Parola P Raoult D Ticks and tick-borne bacterial diseases in humans: an emerging infectious threat Clin Infect Dis 2001 32 897 928 10.1086/319347 11247714

[B2] Jones KE Patel NG Levy MA Storeygard A Balk D Gittleman JL Daszak P Global trends in emerging infectious diseases Nature 2008 451 990 993 10.1038/nature06536 18288193 PMC5960580

[B3] Estrada-Peña A Osácar JJ Pichon B Gray JS Hosts and pathogen detection for immature stages of *Ixodes ricinus* (Acari: Ixodidae) in North-Central Spain Exp Appl Acarol 2005 37 257 268 10.1007/s10493-005-3271-6 16323055

[B4] Swanson SJ Neitzel D Reed KD Belongia EA Coinfections acquired from *Ixodes* ticks Clin Microbiol Rev 2006 19 708 727 10.1128/CMR.00011-06 17041141 PMC1592693

[B5] Medlock JM Hansford KM Bormane A Derdakova M Estrada-Peña A George JC Golovljova I Jaenson TG Jensen JK Jensen PM Kazimirova M Oteo JA Papa A Pfister K Plantard O Randolph SE Rizzoli A Santos-Silva MM Sprong H Vial L Hendrickx G Zeller H Van Bortel W Driving forces for changes in geographical distribution of *Ixodes ricinus* ticks in Europe Parasit Vectors 2013 6 1 10.1186/1756-3305-6-1 23281838 PMC3549795

[B6] Overzier E Pfister K Thiel C Herb I Mahling M Silaghi C *Anaplasma phagocytophilum* in questing *Ixodes ricinus* ticks: comparison of prevalences and partial 16S rRNA gene variants in urban, pasture, and natural habitats Appl Environ Microbiol 2013 79 1730 1734 10.1128/AEM.03300-12 23263964 PMC3591944

[B7] Randolph SE Tick ecology: processes and patterns behind the epidemiological risk posed by Ixodid ticks as vectors Parasitology 2004 129 S37 S65 10.1017/S0031182004004925 15938504

[B8] Carpi G Cagnacci F Neteler M Rizzoli A Tick infestation on roe deer in relation togeographic and remotely sensed climatic variables in a tick-borne encephalitis endemic area Epidemiol Infect 2008 136 1416 1424 18081949 10.1017/S0950268807000039 PMC2870723

[B9] Pugliese A Rosà R Effect of host populations on the intensity of ticks and the prevalence of tick-borne pathogens: how to interpret the results of deer exclosure experiments Parasitology 2008 135 1531 1544 10.1017/S003118200800036X 18442427

[B10] Overzier E Pfister K Thiel C Herb I Mahling M Silaghi C Diversity of *Babesia* and *Rickettsia* species in questing *Ixodes ricinus*: a longitudinal study in urban, pasture, and natural habitats Vector Borne Zoonotic Dis 2013 13 559 564 10.1089/vbz.2012.1278 23697771 PMC3741418

[B11] Overzier E Pfister K Herb I Mahling M Böck G Jr Silaghi C Detection of tick-borne pathogens in roe deer (*Capreolus capreolus*), in questing ticks (*Ixodes ricinus*), and in ticks infesting roe deer in southern Germany Ticks Tick Borne Dis 2013 4 320 328 10.1016/j.ttbdis.2013.01.004 23571115

[B12] Welc-Falęciak R Bajer A Behnke JM Siński E Effects of host diversity and the community composition of Ixodid ticks (Ixodidae) on *Babesia microti* infection Int J Med Microbiol 2008 298 235 242

[B13] Hildebrandt A Franke J Meier F Sachse S Dorn W Straube E The potential role of migratory birds in transmission cycles of *Babesia* spp., *Anaplasma phagocytophilum*, and *Rickettsia* spp Ticks Tick Borne Dis 2010 1 105 107 10.1016/j.ttbdis.2009.12.003 21771516

[B14] Welc-Falęciak R Werszko J Cydzik K Bajer A Michalik J Behnke JM Co-infection and genetic diversity of tick-borne pathogens in roe deer from Poland Vector Borne Zoonotic Dis 2013 13 277 288 10.1089/vbz.2012.1136 23473225 PMC3636586

[B15] Welc-Falęciak R Bajer A Paziewska-Harris A Baumann-Popczyk A Siński E Diversity of *Babesia* in *Ixodes ricinus* ticks in Poland Adv Med Sci 2012 57 364 369 22968337 10.2478/v10039-012-0023-9

[B16] Siński E Bajer A Welc R Pawełczyk A Ogrzewalska M Behnke JM *Babesia microti*: prevalence in wild rodents and *Ixodes ricinus* ticks from the Mazury Lakes District of North-Eastern Poland Int J Med Microbiol 2006 296 137 143 16524774 10.1016/j.ijmm.2006.01.015

[B17] Eremeeva ME Molecular epidemiology of rickettsial diseases in North America Ticks Tick Borne Dis 2012 3 332 337 10.1016/j.ttbdis.2012.10.022 23182271

[B18] Reye AL Stegniy V Mishaeva NP Velhin S Hübschen JM Ignatyev G Muller CP Prevalence of tick-borne pathogens in *Ixodes ricinus* and *Dermacentor reticulatus* ticks from different geographical locations in Belarus PLoS One 2013 8 e54476 10.1371/journal.pone.0054476 23349900 PMC3551763

[B19] Roux V Rydkina E Eremeeva M Raoult D Citrate synthase gene comparison, a new tool for phylogenetic analysis, and its application for the rickettsiae Int J Syst Bacteriol 1997 47 252 261 10.1099/00207713-47-2-252 9103608

[B20] Rar VA Golovljova I *Anaplasma*, *Ehrlichia*, and “*Candidatus* Neoehrlichia” bacteria: pathogenicity, biodiversity, and molecular genetic characteristics, a review Infect Genet Evol 2011 11 1842 1861 10.1016/j.meegid.2011.09.019 21983560

[B21] Bakken JS Dumler JS Human granulocytic ehrlichiosis Clin Infect Dis 2000 31 554 560 10.1086/313948 10987720

[B22] Keesing F Hersh MH Tibbetts M McHenry DJ Duerr S Brunner J Killilea M LoGiudice K Schmidt KA Ostfeld RS Reservoir competence of vertebrate hosts for *Anaplasma phagocytophilum* Emerg Infect Dis 2012 18 2013 2016 10.3201/eid1812.120919 23171835 PMC3557888

[B23] Massung RF Priestley RA Levin ML Route of transmission alters the infectivity of *Anaplasma phagocytophila* in mice Ann N Y Acad Sci 2003 990 494 495 10.1111/j.1749-6632.2003.tb07416.x 12860679

[B24] Petrovec M Bidovec A Sumner JW Nicholson WL Childs JE Avsic-Zupanc T Infection with *Anaplasma phagocytophila* in cervids from Slovenia: evidence of two genotypic lineages Wien Klin Wochenschr 2002 114 641 647 12422618

[B25] Petrovec M Sixl W Schweiger R Mikulasek S Elke L Wüst G Marth E Strasek K Stünzner D Avsic-Zupanc T Infections of wild animals with *Anaplasma phagocytophila* in Austria and the Czech Republic Ann N Y Acad Sci 2003 990 103 106 10.1111/j.1749-6632.2003.tb07345.x 12860608

[B26] von Loewenich FD Baumgarten BU Schröppel K Geissdörfer W Röllinghoff M Bogdan C High diversity of *ankA* sequences of *Anaplasma phagocytophilum* among *Ixodes ricinus* ticks in Germany J Clin Microbiol 2003 41 5033 5040 10.1128/JCM.41.11.5033-5040.2003 14605135 PMC262509

[B27] Kawahara M Rikihisa Y Isogai E Takahashi M Misumi H Suto C Shibata S Zhang C Tsuji M Ultrastructure and phylogenetic analysis of ‘*Candidatus* Neoehrlichia mikurensis’ in the family *Anaplasmataceae*, isolated from wild rats and found in *Ixodes ovatus* ticks Int J Syst Evol Microbiol 2004 54 1837 1843 10.1099/ijs.0.63260-0 15388752

[B28] Li H Jiang J Tang F Sun Y Li Z Zhang W Gong Z Liu K Yang H Liu W Cao W Wide distribution and genetic diversity of “*Candidatus* Neoehrlichia mikurensis” in rodents from China Appl Environ Microbiol 2013 79 1024 1027 10.1128/AEM.02917-12 23183973 PMC3568564

[B29] Vayssier-Taussat M Le Rhun D Buffet JP Maaoui N Galan M Guivier E Charbonnel N Cosson JF *Candidatus* Neoehrlichia mikurensis in bank voles, France Emerg Infect Dis 2012 18 2063 2065 10.3201/eid1812.120846 23171720 PMC3557860

[B30] Fehr JS Bloemberg GV Ritter C Hombach M Lüscher TF Weber R Keller PM Septicemia caused by tick-borne bacterial pathogen *Candidatus* Neoehrlichia mikurensis Emerg Infect Dis 2010 16 1127 1129 10.3201/eid1607.091907 20587186 PMC3358111

[B31] Pekova S Vydra J Kabickova H Frankova S Haugvicova R Mazal O Cmejla R Hardekopf DW Jancuskova T Kozak T *Candidatus* Neoehrlichia mikurensis infection identified in 2 hematooncologic patients: benefit of molecular techniques for rare pathogen detection Diagn Microbiol Infect Dis 2011 69 266 270 10.1016/j.diagmicrobio.2010.10.004 21353949

[B32] von Loewenich FD Geissdörfer W Disqué C Matten J Schett G Sakka SG Bogdan C Detection of “*Candidatus* Neoehrlichia mikurensis” in two patients with severe febrile illnesses: evidence for a European sequence variant J Clin Microbiol 2010 48 2630 2635 10.1128/JCM.00588-10 20519481 PMC2897504

[B33] Welinder-Olsson C Kjellin E Vaht K Jacobsson S Wennerås C First case of human “Candidatus Neoehrlichia mikurensis” infection in a febrile patient with chronic lymphocytic leukemia J Clin Microbiol 2010 48 1956 1959 10.1128/JCM.02423-09 20220155 PMC2863919

[B34] Fertner ME Mølbak L Boye Pihl TP Fomsgaard A Bødker R First detection of tick-borne “*Candidatus* Neoehrlichia mikurensis” in Denmark 2011 Euro Surveill 2012 23 17(8) 22401505

[B35] Richter D Matuschka FR “*Candidatus* Neoehrlichia mikurensis,” *Anaplasma phagocytophilum,* and lyme disease spirochetes in questing european vector ticks and in feeding ticks removed from people J Clin Microbiol 2012 50 943 947 10.1128/JCM.05802-11 22205824 PMC3295140

[B36] Jahfari S Fonville M Hengeveld P Reusken C Scholte EJ Takken W Heyman P Medlock J Heylen D Kleve J Sprong H Prevalence of *Neoehrlichia mikurensis* in ticks and rodents from North-west Europe Parasit Vectors 2012 5 74 10.1186/1756-3305-5-74 22515314 PMC3395572

[B37] Wen B Rikihisa Y Mott J Fuerst PA Kawahara M Suto C *Ehrlichia muris* sp. nov., identified on the basis of 16S rRNA base sequences and serological, morphological, and biological characteristics Int J Syst Bacteriol 1995 45 250 254 10.1099/00207713-45-2-250 7537059

[B38] Spitalská E Boldis V Kostanová Z Kocianová E Stefanidesová K Incidence of various tick-borne microorganisms in rodents and ticks of central Slovakia Acta Virol 2008 52 175 179 18999892

[B39] Subramanian G Sekeyova Z Raoult D Mediannikov O Multiple tick-associated bacteria in *Ixodes ricinus* from Slovakia Ticks Tick Borne Dis 2012 3 406 410 10.1016/j.ttbdis.2012.10.001 23182274

[B40] Bajer A Behnke JM Pawełczyk A Siński E First evidence of *Ehrlichia* sp. in wild *Microtus arvalis* from Poland Acta Parasitologica 1999 44 204 205

[B41] Sumner JW Nicholson WL Massung RF PCR amplification and comparison of nucleotide sequences from the *groESL* heat shock operon of *Ehrlichia* species J Clin Microbiol 1997 35 2087 2092 9230387 10.1128/jcm.35.8.2087-2092.1997 PMC229908

[B42] Massung RF Slater K Owens JH Nicholson WL Mather TN Solberg VB Olson JG Nested PCR assay for detection of granulocytic ehrlichiae J Clin Microbiol 1998 36 1090 1095 9542943 10.1128/jcm.36.4.1090-1095.1998 PMC104695

[B43] Noureddine R Chauvin A Plantard O Lack of genetic structure among Eurasian populations of the tick *Ixodes ricinus* contrasts with marked divergence from north-African populations Int J Parasitol 2011 41 183 192 10.1016/j.ijpara.2010.08.010 20946897

[B44] Tamura K Peterson D Peterson N Stecher G Nei M Kumar S MEGA5: molecular evolutionary genetics analysis using maximum likelihood, evolutionary distance, and maximum parsimony methods Mol Biol Evol 2011 28 2731 2739 10.1093/molbev/msr121 21546353 PMC3203626

[B45] Estrada-Peña A Understanding the relationships between landscape connectivity and abundance of *Ixodes ricinus* ticks Exp Appl Acarol 2002 28 239 248 14570137 10.1023/a:1025362903620

[B46] Dobson AD Taylor JL Randolph SE Tick (*Ixodes ricinus*) abundance and seasonality at recreational sites in the UK: hazards in relation to fine-scale habitat types revealed by complementary sampling methods Ticks Tick Borne Dis 2011 2 67 74 10.1016/j.ttbdis.2011.03.002 21771540

[B47] Schwarz A Maier WA Kistemann T Kampen H Analysis of the distribution of the tick *Ixodes ricinus* L. (Acari: Ixodidae) in a nature reserve of western Germany using Geographic Information Systems Int J Hyg Environ Health 2009 212 87 96 10.1016/j.ijheh.2007.12.001 18262840

[B48] Lindström A Jaenson TG Distribution of the common tick, *Ixodes ricinus* (Acari: Ixodidae), in different vegetation types in southern Sweden J Med Entomol 2003 40 375 378 10.1603/0022-2585-40.4.375 14680099

[B49] James MC Bowman AS Forbes KJ Lewis F McLeod JE Gilbert L Environmental determinants of *Ixodes ricinus* ticks and the incidence of *Borrelia burgdorferi* sensu lato, the agent of Lyme borreliosis, in Scotland Parasitology 2013 140 237 246 10.1017/S003118201200145X 23036286

[B50] Estrada-Peña A Acevedo P Ruiz-Fons F Gortazar C de la Fuente J Evidence of the importance of host habitat use in predicting the dilution effect of wild boar for deer exposure to *Anaplasma* spp PLoS ONE 2008 3 e2999 10.1371/journal.pone.0002999 18714379 PMC2500193

[B51] Reye AL Hübschen JM Sausy A Muller CP Prevalence and seasonality of tick-borne pathogens in questing Ixodes ricinus ticks from Luxembourg Appl Environ Microbiol 2010 76 2923 2931 10.1128/AEM.03061-09 20228110 PMC2863427

[B52] Cotté V Bonnet S Cote M Vayssier-Taussat M Prevalence of five pathogenic agents in questing *Ixodes ricinus* ticks from western France Vector Borne Zoonotic Dis 2010 10 723 730 10.1089/vbz.2009.0066 20020814

[B53] Rymaszewska A Piotrowski M Use of DNA sequences for *Rickettsia* identification in *Ixodes ricinus* ticks: the first detection of *Rickettsia monacensis* in Poland Microbes Infect 2013 15 140 146 10.1016/j.micinf.2012.11.005 23178758

[B54] Venclikova K Rudolf I Mendel J Betasova L Hubalek Z Rickettsiae in questing Ixodes ricinus ticks in the Czech Republic Ticks Tick Borne Dis 2013 doi:10.1016/j.ttbdis.2013.09.008 10.1016/j.ttbdis.2013.09.008 24252265

[B55] Jado I Oteo JA Aldámiz M Gil H Escudero R Ibarra V Portu J Portillo A Lezaun MJ García-Amil C Rodríguez-Moreno I Anda P *Rickettsia monacensis* and human disease, Spain Emerg Infect Dis 2007 13 1405 1407 10.3201/eid1309.060186 18252123 PMC2857266

[B56] Tijsse-Klasen E Sprong H Pandak N Co-infection of *Borrelia burgdorferi* sensu lato and *Rickettsia* species in ticks and in an erythema migrans patient Parasit Vectors 2013 6 347 10.1186/1756-3305-6-347 24326096 PMC3878868

[B57] Simser JA Palmer AT Fingerle V Wilske B Kurtti TJ Munderloh UG *Rickettsia monacensis* sp. nov., a spotted fever group *Rickettsia*, from ticks (*Ixodes ricinus*) collected in a European city park Appl Environ Microbiol 2002 68 4559 4566 10.1128/AEM.68.9.4559-4566.2002 12200314 PMC124077

[B58] Ionita M Mitrea IL Pfister K Hamel D Silaghi C Molecular evidence for bacterial and protozoan pathogens in hard ticks from Romania Vet Parasitol 2013 196 71 76 10.1016/j.vetpar.2013.01.016 23428204

[B59] Wójcik-Fatla A Szymańska J Wdowiak L Buczek A Dutkiewicz J Coincidence of three pathogens (*Borrelia burgdorferi* sensu lato, *Anaplasma phagocytophilum* and *Babesia microti*) in *Ixodes ricinus* ticks in the Lublin macroregion Ann Agric Environ Med 2009 16 151 158 19630205

[B60] Liz JS Anderes L Sumner JW Massung RF Gern L Rutti B Brossard M PCR detection of granulocytic ehrlichiae in Ixodes ricinus ticks and wild small mammals in western Switzerland J Clin Microbiol 2000 38 1002 1007 10698987 10.1128/jcm.38.3.1002-1007.2000 PMC86323

[B61] Katargina O Geller J Alekseev A Dubinina H Efremova G Mishaeva N Vasilenko V Kuznetsova T Järvekülg L Vene S Lundkvist A Golovljova I Identification of *Anaplasma phagocytophilum* in tick populations in Estonia, the European part of Russia and Belarus Clin Microbiol Infect 2012 18 40 46 21199155 10.1111/j.1469-0691.2010.03457.x

[B62] Cinco M Padovan D Murgia R Maroli M Frusteri L Heldtander M Johansson KE Engvall EO Coexistence of *Ehrlichia phagocytophila* and *Borrelia burgdorferi* sensu lato in *Ixodes ricinus* ticks from Italy as determined by 16S rRNA gene sequencing J Clin Microbiol 1997 35 3365 3366 9399564 10.1128/jcm.35.12.3365-3366.1997 PMC230192

[B63] Portillo A Pérez-Martínez L Santibáñez S Santibáñez P Palomar AM Oteo JA *Anaplasma* spp. in wild mammals and *Ixodes ricinus* from the north of Spain Vector Borne Zoonotic Dis 2011 11 3 8 10.1089/vbz.2009.0214 20528172

[B64] Lommano E Bertaiola L Dupasquier C Gern L Infections and coinfections of questing *Ixodes ricinus* ticks by emerging zoonotic pathogens in Western Switzerland Appl Environ Microbiol 2012 78 4606 4612 10.1128/AEM.07961-11 22522688 PMC3370488

[B65] Sprong H Trentelman J Seemann I Grubhoffer L Rego RO Hajdušek O Kopáček P Síma R Nijhof AM Anguita J Winter P Rotter B Havlíková S Klempa B Schetters TP Hovius JW ANTIDotE: anti-tick vaccines to prevent tick-borne diseases in Europe Parasit Vectors 2014 7 77 doi:10.1186/1756-3305-7-77 10.1186/1756-3305-7-77 24559082 PMC3933510

